# Healthcare Worker’s Satisfaction Assessment for a Healthcare Adverse Event Reporting Framework and the Management Approach for Such Reporting in the Emergency Department of Rural Government Hospitals

**DOI:** 10.7759/cureus.62905

**Published:** 2024-06-22

**Authors:** Jyotsana Singh, Anita Choudhary, Somendra P Singh, Pankaj Singh

**Affiliations:** 1 Department of Management, NIMS University Rajasthan, Jaipur, IND; 2 Department of Management and Commerce, NIMS University Rajasthan, Jaipur, IND; 3 Department of General Surgery, Uttar Pradesh University of Medical Sciences, Etawah, IND; 4 Department of Anaesthesia, Uttar Pradesh University of Medical Sciences, Etawah, IND

**Keywords:** emergency department, framework, healthcare adverse event, management approach, nonparametric, ordinal, patient safety, reporting, rural, satisfaction

## Abstract

Background: The healthcare adverse event (HAE) reporting framework is more than just a tool. It is a crucial pillar in our pursuit of patient safety, quality improvement, trust maintenance, regulatory compliance, and ethical standards in healthcare delivery.

Aim: To assess healthcare workers' satisfaction with the HAE reporting framework and the management approach towards such reporting in the emergency department of rural government hospitals by conducting a satisfaction survey.

Materials and method: This prospective observational research was conducted in the Department of Emergency Medicine of the Uttar Pradesh University of Medical Sciences, Saifai, and adjoining district hospitals from November 2023 to January 2024. The study involved 320 healthcare professionals working in the emergency department. The quantitative survey research used a questionnaire and a quality Likert scale response. The data were analyzed on an ordinal measurement scale using nonparametric statistical methods. The sample data were analyzed using frequency tables, percentage pie charts, and comparison bar graphs. In nonparametric statistical tests, the one-sample Wilcoxon signed rank test was used to infer the population's central tendency, and the Kruskal-Wallis test was used to make inferences about the population categories.

Results: The satisfaction of healthcare professionals with the HAE reporting framework and the management approach was diverse. When surveyed about the HAE reporting framework in the emergency department, out of the 320 healthcare professionals, 50% (161) expressed dissatisfaction, 47% (149) were satisfied, and 3% (10) did not comment. Paramedics were most dissatisfied (61% of 133). When asked about the management approach while dealing with such reporting, 50% (159) were satisfied, 46% (147) were unsatisfied, and 4% (14) did not comment. On comparing professions, 43% (29) of 33 doctors and 62% (83) of 133 paramedics were unsatisfied, giving a poor response. Additionally, 61% (72) of the 119 nursing staff were satisfied. The non-parametric inferential tests yielded a p-value of less than 0.001 for both questions, indicating a notable difference in the population's response to the HAE reporting framework and management approach. On pairwise comparison, there was a significant difference in perception (p<0.001) between the occupation groups, except for doctors and paramedics (p = 0.638) in the HAE reporting framework.

Conclusion: By encouraging reporting, standardizing processes, analyzing incidents thoroughly, and using data-driven insights to inform improvement efforts, healthcare organizations can enhance patient safety, improve quality of care, and prevent future adverse events. The management approach to HAE reporting involves fostering a culture of safety and transparency, implementing standardized reporting systems, providing education and training to healthcare staff, establishing feedback mechanisms, conducting robust analysis of reported events, promoting continuous improvement, and ensuring transparency and accountability.

## Introduction

Healthcare adverse events (HAE) refer to unintended injuries or complications resulting from medical care. HAE, though challenging, present an opportunity for significant improvement in patient safety and quality of care. These events, which can occur at any stage of healthcare delivery, from diagnosis to treatment and follow-up, are often the result of errors in medication administration, surgical procedures, or miscommunication among healthcare providers [1]. They can lead to patient harm, prolonged hospital stays, or even fatalities. However, by understanding their causes and consequences, healthcare organizations can employ various strategies, such as error reporting systems and quality improvement initiatives, to prevent and mitigate adverse events and enhance patient outcomes [2].

Emergency care can be defined as the delivery of time-sensitive interventions needed to avoid death and disability, for which delays of hours can worsen the prognosis or render care less effective [3]. The most essential part of emergency care includes timely access and acute care delivery for critically ill and injured patients. Healthcare workers are liable and can be sued for failing to provide care promptly if any conduct contributes to medical malpractice. Medical malpractice occurs when the negligence of a healthcare professional causes injury to a patient with whom they have had a professional relationship [4].

Several factors contribute to HAE. Human error is a significant factor, as mistakes made by healthcare providers during diagnosis, treatment, or medication administration can lead to adverse events. Communication breakdowns among healthcare team members or between providers and patients can also contribute to errors. Equipment malfunctions, such as technical failures or inadequacies in medical devices or equipment, are another potential cause of adverse events. Systemic issues within healthcare systems, such as inadequate staffing, poor protocols, or insufficient training, can increase the likelihood of adverse events. Patient-related factors, including complex medical conditions, non-compliance with treatment, allergies, and environmental factors related to the healthcare environment, such as overcrowding or inadequate facilities, may also play a role in contributing to adverse events. Identifying and addressing these causes is essential for preventing and mitigating adverse events in healthcare settings [5].

A reporting framework in healthcare refers to a structured system for documenting and analyzing adverse events, near misses, and other safety incidents within healthcare organizations [6]. It typically involves standardized forms or electronic reporting systems where healthcare professionals can submit incident reports confidentially [7]. These reports are then analyzed to identify patterns, root causes, and trends to improve patient safety and prevent future occurrences. Effective reporting frameworks promote transparency, accountability, and a culture of learning from mistakes. They often include mechanisms for feedback, communication, and continuous improvement to enhance overall healthcare quality and patient outcomes.

A comprehensive management approach to HAE reporting recognizes the crucial role of healthcare professionals. First, it is essential to promote a reporting culture where healthcare professionals feel safe and empowered to report adverse events without fear of retribution. Emphasizing the importance of their role in reporting for learning and improvement is crucial in this regard. Secondly, implementing standardized reporting systems with uniform reporting forms and procedures across healthcare facilities ensures data collection and analysis consistency. This step helps streamline the reporting process and ensures that all necessary information is captured. Next, providing education and training to healthcare staff on the importance of adverse event reporting, recognizing and reporting incidents, and understanding their potential impact on patient safety is essential. This ensures that all staff members are aware of the significance of reporting and are equipped with the necessary knowledge and skills to do so effectively.

Establishing clear feedback mechanisms is a crucial part of a comprehensive management approach to HAE reporting. This involves creating channels for providing reporters with feedback about the outcomes of their reports and sharing insights gained from incident analysis with relevant stakeholders. This step helps in closing the loop and ensuring that reporters are kept informed about the impact of their reports, fostering a sense of engagement and continuous learning. Furthermore, allocating resources and expertise to thoroughly analyze reported events is important to identify root causes, contributing factors, and trends. Using data-driven insights to inform quality improvement initiatives is a key aspect of this step. Continuous improvement is an ongoing process that involves implementing strategies for ongoing improvement, such as updating protocols and procedures, conducting targeted training programs, and monitoring the effectiveness of interventions. Lastly, fostering transparency by sharing lessons from adverse events with relevant stakeholders and holding individuals and systems accountable for addressing identified issues is crucial. This step helps in creating a culture of accountability and continuous learning within the healthcare system.

Healthcare organizations can enhance patient safety, improve quality of care, and prevent future incidents by adopting a proactive and systematic approach to adverse event reporting management [8]. Although current risk identification methods in healthcare have strengths and limitations, it is an open question whether they have been implemented optimally and how well they have been integrated to provide a complete picture of risk within complex healthcare systems. Gaps in the theoretical and conceptual underpinning of safety climate and the evidence base for its practical application in healthcare remain.

A review of the evidence on emergency medical systems as applicable to low- and middle-income countries reveals many gaps in global knowledge [9]. There is a need to better understand the epidemiology of conditions that emergency systems may address in these countries and which interventions may adequately address them. Unfortunately, while several studies have been carried out that have explored the parameters of patient satisfaction in tertiary care centers and large hospitals in India, very little attention has been paid to studies of patient satisfaction at rural health institutions [10]. This research looks forward to assessing the satisfaction of healthcare professionals with the healthcare safety measures in the emergency departments of rural government hospitals.

## Materials and methods

This research was conducted in the emergency department of a rural government hospital, Uttar Pradesh University of Medical Sciences (UPUMS) Saifai, Etawah, as well as in the emergency rooms of surrounding district hospitals in Uttar Pradesh, India, from November 2023 to January 2024. The population of interest was the healthcare workers in the emergency department during the research time frame. The inclusion criteria were the doctors, paramedics, and nursing staff. Internship students and clerical staff were excluded from the study. The calculated sample size for 500 healthcare workers with a 5% margin of error and 95% confidence interval using Slovin's formula was 237.

Given the purpose and the parameters, the chosen approach was a quantitative research method that was descriptive and comparative in nature. A written questionnaire survey was used to gather data within a specified time. The independent variables were the HAE reporting framework and the hospitals' management approach to such reporting. The dependent variable was the healthcare workers’ quality perception score. The survey instruments were closed-ended, structured, and validated questionnaires, and the responses were evaluated using a quality Likert scale score (appendices). Likert scale items were no comment, very poor, poor, good, and excellent.

The idea behind adding 'no comment' to the Likert scale items was to allow respondents to indicate that either they did not have an opinion on a particular issue or did not have any knowledge about the subject mentioned in the questionnaire. The 'No comment' option also gave a sense of neutrality to the Likert scale survey [11].

Although each option was labeled using numbers, the numerals only indicated orders, rank, and score. This does not necessarily imply that the distance between two adjacent options was equal. The distance between the categories was uneven or unknown. Thus, the scale utilized adhered to the measurements of an ordinal categorical scale. Permissible transformations for ordinal Likert scale data are monotonic transformations or positive linear transformations, but not one-to-one substitutions.

The questionnaire was tested in a pilot study of 30 healthcare workers for reliability of responses by test-retest and regression analysis ordinal Omega (0.72) and validity of the questionnaire by coefficient correlation Pearson (0.6). The expert institutional committee validated the questionnaire. After approval from the scientific and ethical committee, a survey was conducted, and the responses were collected from 320 healthcare workers. The survey included 68 doctors, 119 nursing staff, and 133 paramedics.

The data collected were entered into an Excel spreadsheet (Microsoft® Corp., Redmond, WA). Data entry was done by asking one person to read the values while another entered the data. Having one person read and enter data is highly prone to error. The data were then transferred to the data analytics software tool Statistical Product and Service Solutions (SPSS, version 29; IBM SPSS Statistics for Windows, Armonk, NY) for further data cleansing, coding of the various variables, and statistical analysis. Analyses of the sample data were done with the help of frequency tables, percentage pie charts, and comparison bar graphs. Considering the ordinal scale of responses, median and mode were taken to measure central tendency and skewness for the deviation from the normal distribution. The normality of the distribution was also checked with the Kolmogorov-Smirnov test (K-S test) and the Shapiro-Wilk test (S-W test). In both tests, the p-value was < 0.001, which was less than the significant p-value of 0.05. Thus, the distribution was not normal.

Nonparametric statistical tests were used to make inferences about the population. Parametric tests only work with normally distributed data. One potential problem with using parametric methods for ordinal Likert data is the normality assumption. In contrast, non-parametric tests do not make this type of assumption about the shape of the population from which the study data has been drawn. If a parametric test is used on nonparametric data, then this could trick the test into seeing a significant effect when there is not one. This is very dangerous: ‘the type 1 error’ or ‘false positive’. A nonparametric test for parametric data could reduce the chance of seeing a significant effect when there is one. This is not ideal: a ‘type 2 error’ or a missed opportunity. The type 2 error is statistically the least dangerous of these two errors. Nonparametric tests are less powerful than parametric tests and usually require a large sample size to have the same power as the parametric test to find the difference between groups when the difference actually exists.

The nonparametric tests used were a one-sample Wilcoxon signed rank test to infer the population's central tendency and the Kruskal-Wallis test to compare different demographic variables such as occupation in the population. When comparing two independent groups, it is important to test the assumption of homogeneity of variance using Levene’s Test for Equality of Variances. If the p-value > 0.05, the assumption was met, and the test results were used to infer differences in medians. If the p-value < 0.05, the assumption was violated, and the results indicated the mean rank of the two populations. The Kruskal-Wallis statistical test compared a hypothesis regarding the equality of central tendency (medians/mean tanks) and a pairwise comparison of three professions.

## Results

The data analysis process entails the interpretation of data through analytical and logical reasoning to identify patterns, relationships, or trends within a specific population. In this case, we are focusing on healthcare workers' quality perceptions of safety measures in the emergency department of a rural government hospital. Additionally, we aim to examine variations in quality perception based on demographic variables such as profession.

The process involves several important steps. Initially, we will use descriptive statistics such as tables, pie charts, and bar graphs to gain a comprehensive understanding of the sample data. This will allow us to determine the central tendency, measure of dispersion, and data distribution. Subsequently, it is crucial to evaluate the normality of the data distribution to determine the most suitable inferential statistics for the entire population. Finally, once all assumptions and criteria are satisfied, we can apply inferential statistical tests to derive meaningful conclusions about the population of interest from the data.

Descriptive statistics

Healthcare Workers’ Quality Perception of the HAE Reporting Framework

**Figure 1:**
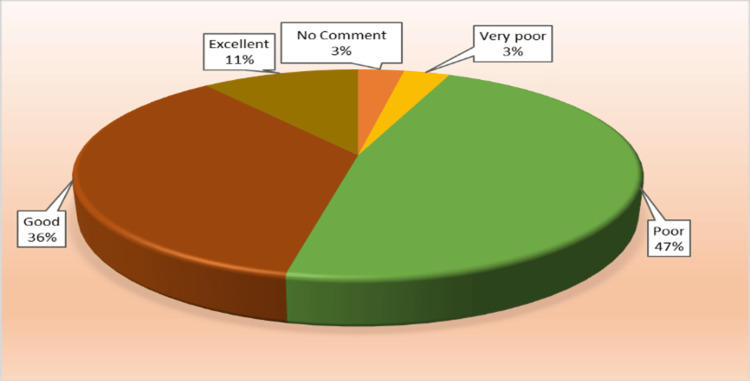
Pie chart of the healthcare adverse event (HAE) reporting framework

Table 1 shows the frequency or number of observations and their relative percentage, and Figure 1 shows the relative distribution percentage. Out of 320 healthcare workers, 50% (151+10) expressed dissatisfaction with the HAE reporting framework. Not far behind, 47% (149) were satisfied with the measures. The median was 3 (middle of the cumulative frequency), and the mode was 3 (poor response).

**Table 1:** Frequency distribution table of the healthcare adverse event reporting framework n = number of respondents, HAE = healthcare adverse event

HAE Reporting framework		Healthcare workers		Professions					
Quality Perception	Score	No. of Respondents		Doctors		Nursing Staff		Paramedics	
		n	%	n	%	n	%	n	%
No Comment	1	10	3.1	8	11.8	1	0.8	1	0.8
Very Poor	2	10	3.1	7	10.3	1	0.8	2	1.5
Poor	3	151	47.2	27	39.7	43	36.1	81	60.3
Good	4	115	35.9	23	33.8	50	42.0	42	31.6
Excellent	5	34	10.6	3	4.4	24	20.3	7	5.3
TOTAL		320		68		119		133	

**Figure 2:**
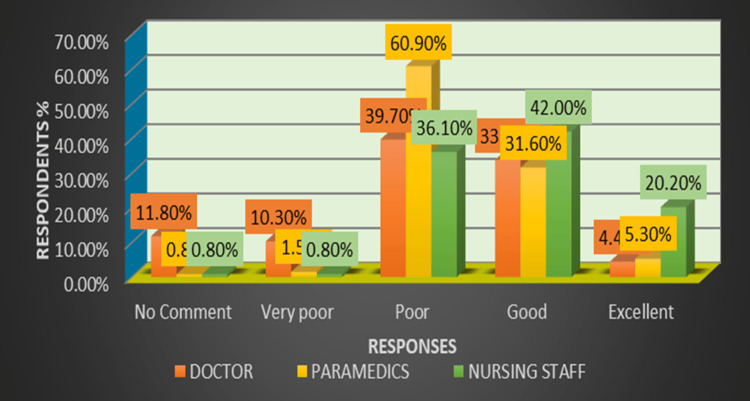
Bar graph showing a comparison of the responses for healthcare adverse event (HAE) reporting framework in three healthcare professions

Table 1 and Figure 2 show that the primary response for the doctors (68) was poor (40%, 27); for the nursing staff (119), it was good (42%, 50); and for the paramedics (133), it was a poor response (61%, 81) when asked about the HAE reporting framework in the emergency department.

Healthcare Workers’ Quality Perception of Management's Approach Towards HAE Reporting

**Figure 3:**
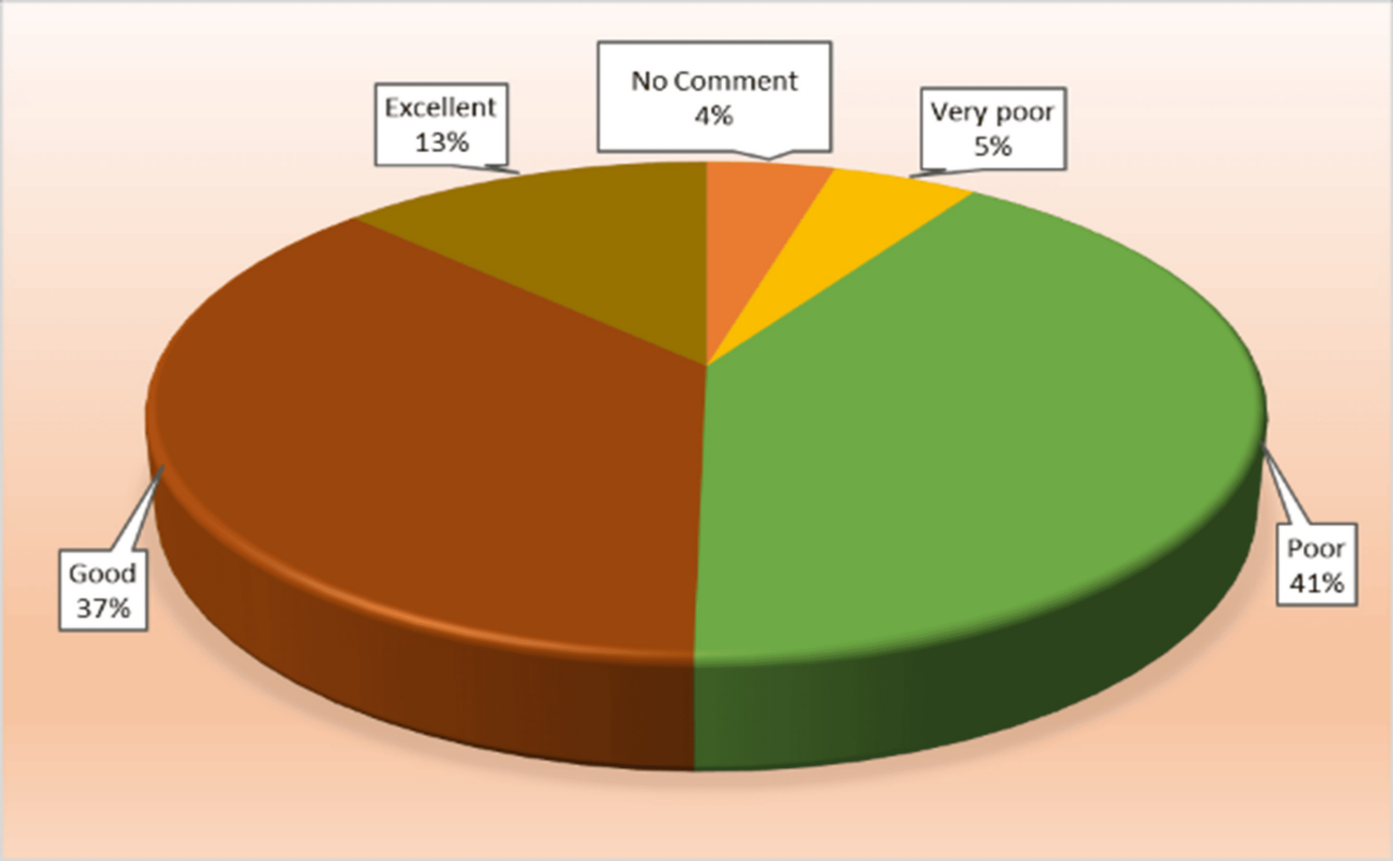
Pie chart of the management approach towards healthcare adverse event (HAE) reporting

Table 2 and Figure 3 show the relative frequency distribution of the responses. Moreover, 50% (159) of the total healthcare workers were satisfied with the hospital's management approach, while 46% (147) were unsatisfied. The median and the mode were poor (3) responses.

**Table 2:** Frequency distribution table of the management approach towards healthcare adverse even (HAE) reporting n = number of respondents

Management Approach		Healthcare Workers		Professions					
Quality Perception	Score	No. of Respondents		Doctors		Nursing Staff		Paramedics	
		n	%	n	%	n	%	n	%
No Comment	1	14	4.4	13	19.1	1	0.8	0	0.0
Very Poor	2	16	5.0	15	22.1	0	0.0	1	0.8
Poor	3	131	40.9	29	42.6	19	16.0	83	62.4
Good	4	118	36.9	9	13.2	72	60.5	37	27.8
Excellent	5	41	12.8	2	2.9	27	22.7	12	9
TOTAL		320	100.0	68	100	119	100	133	100

**Figure 4:**
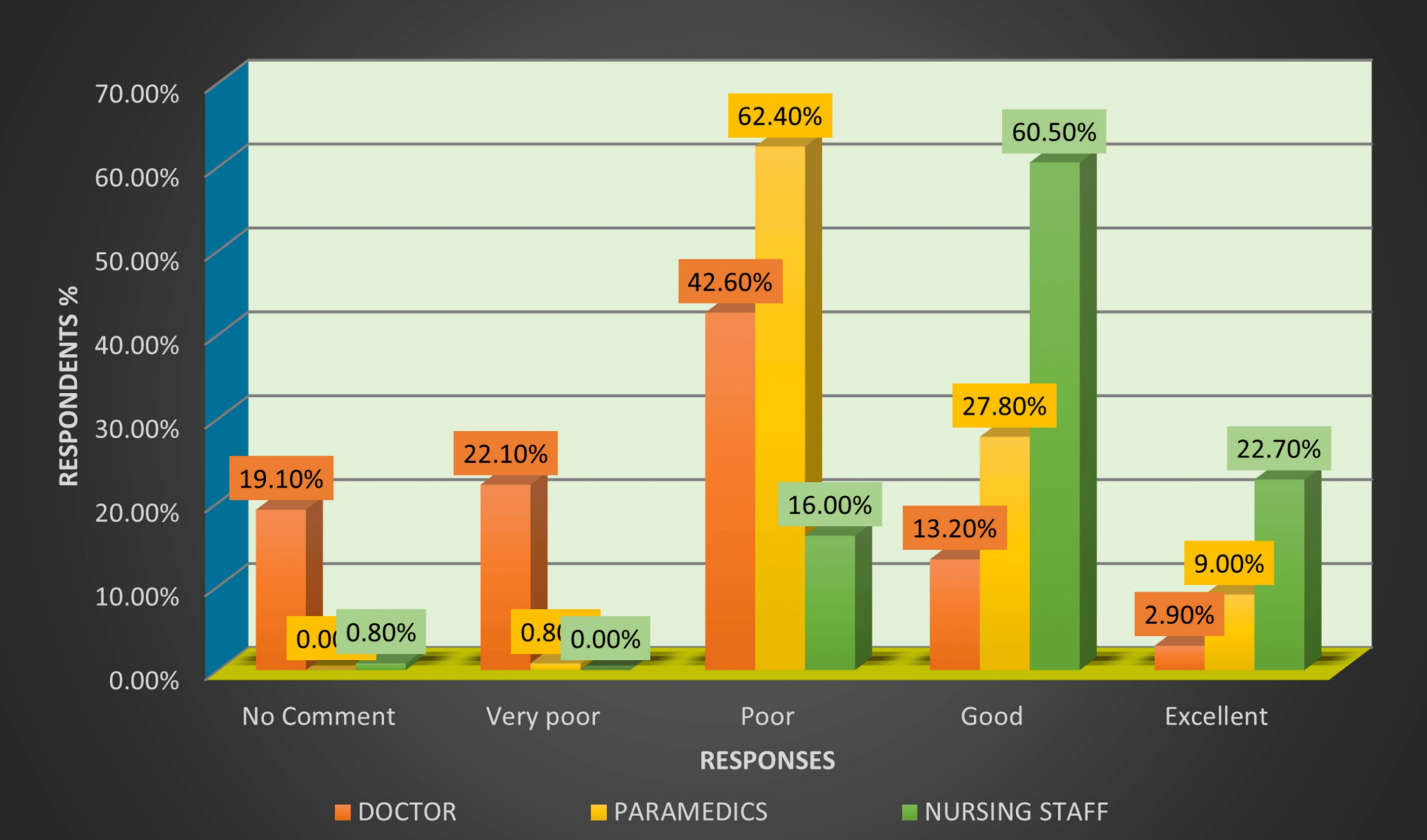
Bar graph showing a comparison of the responses for management approach towards healthcare adverse event (HAE) reporting framework in three healthcare professions

Table 2 and Figure 4 show that the primary response for the doctors was poor (43%, 29); the nursing staff said good (60%, 72); and, for the paramedics, it was a poor response (62%, 83) when asked about the management approach to HAE reporting in the emergency department.

Inferential statistics

Test for Normality of Distribution

Skewness: For the HAE reporting framework, there was a negative skewness of 0.339. The distribution was left-tailed. More values were on the right side of the distribution. For the management approach, there was a negative skewness of 0.487. Left-tailed distribution. Here also, more values were on the right side of the distribution.

The p-value in the K-S and S-W tests was < 0.001 (Table 3) for both the HAE reporting framework and the management approach to such reporting, which was less than the significant 0.05. Thus, the distribution deviated significantly from the normal distribution. We used nonparametric statistical tests to make inferences about the population.

**Table 3:** Tests of normality distribution p-value < 0.05 is considered significant (Sig.), df = degree of freedom, HAE = healthcare adverse event

		Kolmogorov-Smirnov^a^			Shapiro-Wilk		
		Statistic	df	Sig.	Statistic	df	Sig.
HEA reporting framework	Healthcare Professionals	0.249	320	< 0.001	0.842	320	< 0.001
	Doctor	0.187	68	< 0.001	0.873	68	< 0.001
	Paramedics	0.348	133	< 0.001	0.755	133	< 0.001
	Nursing Staff	0.315	119	< 0.001	0.788	119	< 0.001
Management approach	Healthcare Professionals	0.207	320	< 0.001	0.868	320	< 0.001
	Doctor	0.242	68	< 0.001	0.891	68	< 0.001
	Paramedics	0.382	133	< 0.001	0.710	133	< 0.001
	Nursing Staff	0.307	119	< 0.001	0.780	119	< 0.001
a. Lilliefors Significance Correction							

Inference of Central Tendency in the Population

A one-sample Wilcoxon signed rank test yielded a p-value < 0.001 (Table 4) for both questions, which is lower than the significance level of 0.05. Consequently, we rejected the null hypothesis. This indicates that the central tendency of the population's response to the HAE reporting framework and the management approach for such reporting differs from what we previously assumed to be a good response. The test was two-sided and statistically significant, so the responses can be both-sided.

**Table 4:** Nonparametric one-sample Wilcoxon signed rank test p-value < 0.05 significant (Sig.), HAE = healthcare adverse event

	Null Hypothesis	Test	Sig.^a,b^	Decision
1	The median of the HEA reporting framework equals 4.	One-Sample Wilcoxon Signed Rank Test	< 0.001	Reject the null hypothesis.
2	The median of the Management approach equals 4.	One-Sample Wilcoxon Signed Rank Test	< 0.001	Reject the null hypothesis.
a. The significance level is 0.050.				
b. Asymptotic significance is displayed.				

Comparison of the Quality Perception of the Responses of the Doctors, Nursing Staff, and Paramedics

**Figure 5:**
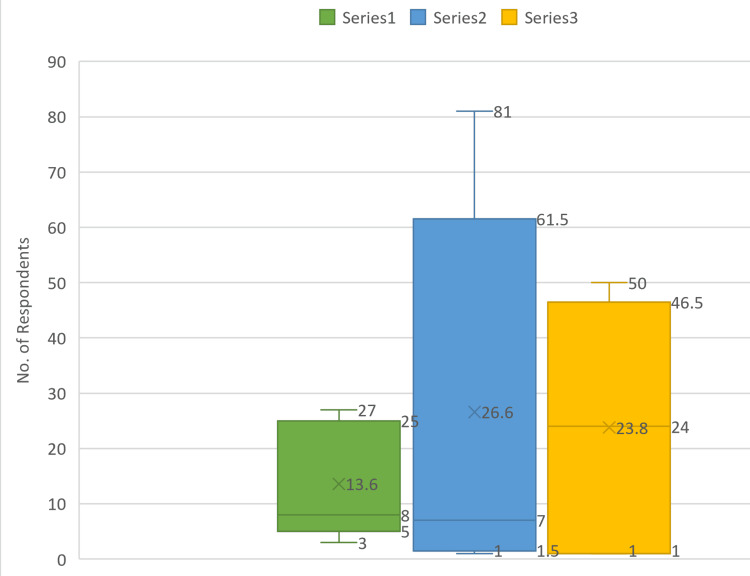
Box plot comparing the spread of data for different professions Series 1: Doctors, Series 2: Paramedics, Series 3: Nursing staff

Homogeneity of variance: The distribution of responses for the HAE reporting framework among different professions was not the same (Figure 5).

**Figure 6:**
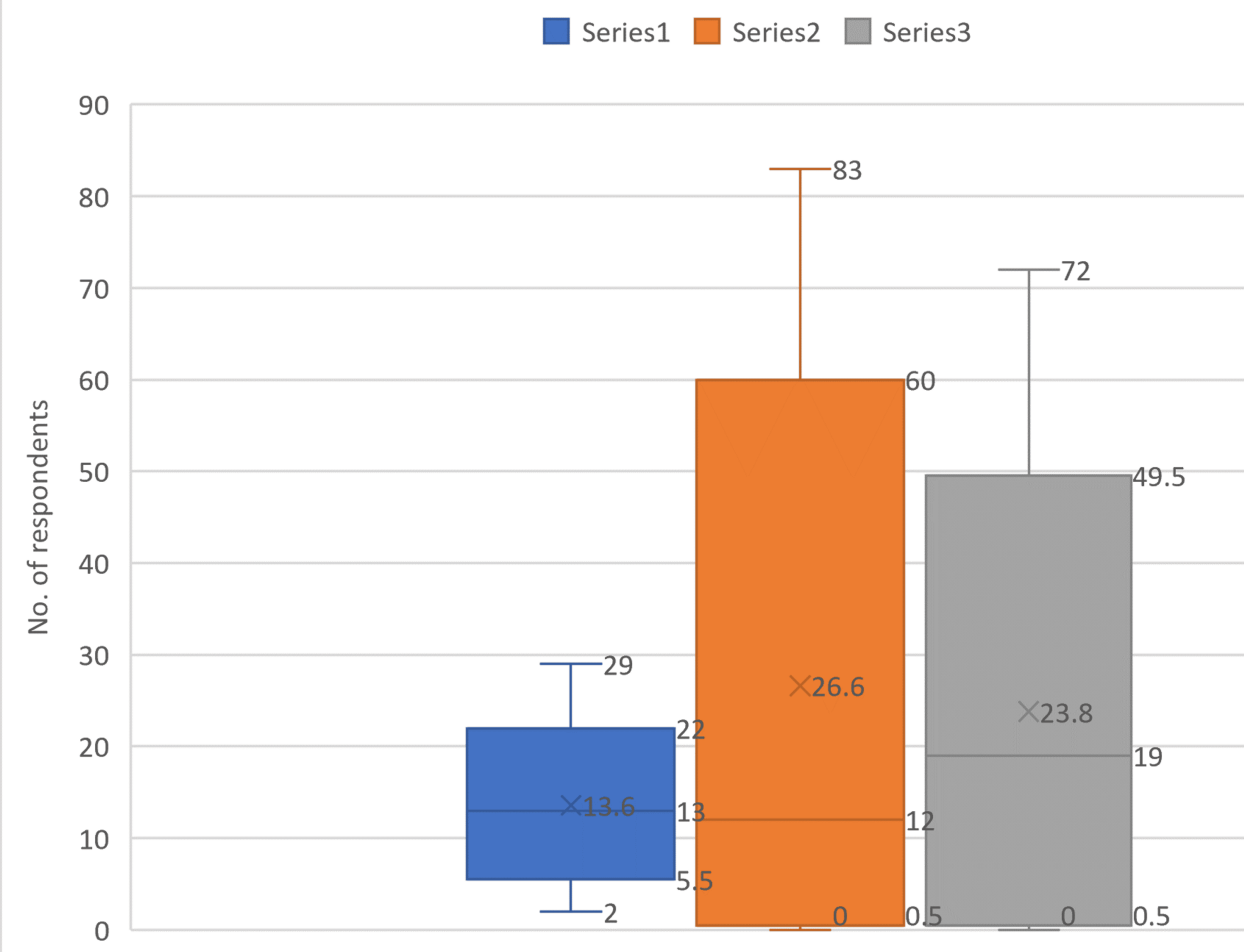
Box plot comparing the spread of data for different professions Series 1: Doctors, Series 2: Paramedics, Series 3: Nursing staff

The distribution of responses for the management approach towards HAE reporting was not the same (Figure 6).

In the homogeneity of variance test (Table 5), the p-values (adjusted for median and degrees of freedom) were found to be < 0.001 for the HAE reporting framework and 0.003 for the management approach, indicating significant heterogeneity as both values were less than the critical threshold of 0.05. Consequently, we proceeded to use the mean ranks to compare the distribution of responses across the three professions within the population.

**Table 5:** Levene's test for homogeneity of variance for occupation p-value < 0.05 significant df = degree of freedom, HAE = healthcare adverse event

		Levene Statistic	df1	df2	Sig.
HAE reporting framework	Based on Mean	5.492	2	317	0.005
	Based on Median	6.090	2	317	0.003
	Based on the Median and with adjusted df	6.090	2	305.121	0.003
	Based on trimmed mean	6.025	2	317	0.003
Management approach	Based on Mean	19.086	2	317	< 0.001
	Based on Median	8.200	2	317	< 0.001
	Based on the Median and with adjusted df	8.200	2	293.966	< 0.001
	Based on trimmed mean	18.272	2	317	< 0.001

Non-parametric Test: Comparing Three Professions

The independent sample Kruskal-Wallis test yielded a significant p-value of less than 0.001 (Table 6) for the HAE reporting framework and management approach towards HAE reporting, which was below the significance level of 0.05. As a result, we rejected the null hypothesis. The distribution of the mean ranks of the responses indicates that the perception of the quality of healthcare worker measures in the emergency department varies across the categories of healthcare professions in the population.

**Table 6:** Hypothesis test summary for the comparison of three professions p-value < 0.05 significant (Sig.), HAE = healthcare adverse event

	Null Hypothesis	Test	Sig.^a,b^	Decision
1	The distribution of the HAE reporting framework is the same across categories of profession.	Independent-Samples Kruskal-Wallis Test	< 0.001	Reject the null hypothesis
2	The distribution of the management approach is the same across profession categories.	Independent-Samples Kruskal-Wallis Test	< 0.001	Reject the null hypothesis
a. The significance level is 0.050.				
b. Asymptotic significance is displayed.				

In Table 7, a pairwise comparison of occupations revealed a significant difference in perception (p < 0.001) between the occupation groups, with the exception of doctors and paramedics (p = 0.638) in relation to the HAE reporting framework. The responses of doctors and paramedics to the HAE reporting framework were found to be almost identical.

**Table 7:** Pairwise comparisons of professions Each row tests the null hypothesis that the Sample 1 and Sample 2 distributions are the same. Asymptotic significances (2-sided tests) are displayed. The significance (Sig.) level p-value is 0.050. a. Significance values have been adjusted by the Bonferroni correction for multiple tests.

Sample 1-Sample 2	Test Statistic	Std. Error	Std. Test Statistic	Sig.	Adj. Sig.^a^
Healthcare professional safety measures					
Healthcare adverse event reporting framework					
Doctor-Paramedics	-15.824	12.696	-1.246	0.213	0.638
Doctor-Nursing Staff	-61.088	12.946	-4.719	< 0.001	0.000
Paramedics-Nursing Staff	-45.264	10.746	-4.212	< 0.001	0.000
Management approach to HAE reporting					
Doctor-Paramedics	-63.143	12.931	-4.883	< 0.001	0.000
Doctor-Nursing Staff	-131.524	13.186	-9.975	< 0.001	0.000
Paramedics-Nursing Staff	-68.381	10.945	-6.248	< 0.001	0.000

## Discussion

Healthcare systems can be divided into four main domains: healthcare workers, patients receiving health care, healthcare delivery processes, feedback, and continuous improvement methods [12]. The HAE reporting framework is crucial to ensuring patient safety and improving the quality of care provided. To facilitate an effective reporting culture within a healthcare organization, healthcare professionals must be encouraged to report incidents, near misses, and adverse events without fear of punitive measures. In this regard, it is essential to identify and address the barriers that hinder healthcare professionals from reporting such events, such as a lack of awareness, fear of reprisal, or the perception that reporting is time-consuming.

Several studies have been conducted to assess healthcare professionals' compliance with hospital policies. One study by Kottapalli et al. in 2023 [13] aimed to identify staff education requirements on safe injection and infusion practices. Another study by Liu et al. in 2023 [14] looked back over five years in a pediatric hospital to assess the occurrence and features of medication errors. In a retrospective cohort study conducted by Mira et al. in 2023 [15] in primary care settings in Spain, the frequency and severity of adverse events that occur when do-not-do (DND) recommendations are ignored were determined. Additionally, patient identification errors during inter-hospital transfers were studied by Suclupe et al. in 2023 [16] and Hughes in 2023 [17]. It was discovered that avoidable incidents causing patient harm persist despite implementing preventive measures. Furthermore, a bibliometric analysis was conducted in 2022 by Ünal et al. [18] to investigate and evaluate the global research on medical error reporting and reporting systems. The study revealed that several underdeveloped or developing countries have insufficient publications and cross-country collaborations on error reporting. Another retrospective bibliometric analysis conducted by Yeung et al. in 2022 [19] aimed to quantitatively analyze the scientific literature related to legal regulations for patient safety.

Simsekler et al. [20] devised a system-based approach called the risk identification (RID) framework to identify new risks in real-world healthcare settings proactively. To address the issue of communication breakdowns resulting in adverse events in healthcare, clinical team training (CTT) was developed by Schwartz et al. [21]. Additionally, several studies investigated the impact of healthcare attributes, such as nursing care and physician care, on overall healthcare satisfaction [22].

In India, particularly in rural areas, one of the biggest challenges to healthcare safety is the need for more policies, regulations, standard operating procedures, transparency and accountability, and a structured monitoring system. Affective emergency medical systems provide timely medical care to prevent death or disability. Most emergency departments in universities and government hospitals do not match up to the 'emergency department categorization standards' proposed by the Society of Academic Emergency Medicine (SAEM) [23]. Another primary concern was the lack of emergency protocols, standard operating procedures (SOPs), and guidelines. There is a paucity of courses and learning materials, particularly regarding emergency medicine safety research. As a neglected topic, emergency medical systems are part of the 10/90 gap in health research, whereby less than 10% of global research investment is spent on problems affecting 90% of the world’s population [24]. These gaps reflect the need for a more systematic analysis of the areas towards which research investments should be directed so that systems can be based on credible evidence.

In this study, satisfaction and dissatisfaction scores were almost the same, and this typically indicated a polarised response. This meant that the participants had a split opinion, with some finding the implemented measures positive and others negative. Such results might suggest underlying issues or inconsistencies in healthcare services that cater well to some preferences but fail others, mainly paramedics. It could also highlight areas for improvement or the need for a more targeted approach to address specific concerns and enhance overall satisfaction.

HAE reporting is crucial for improving patient safety and care quality, but this process has several challenges and flaws. Underreporting is a common issue due to fear of consequences, fear of blame, reprisal, or a lack of confidence in the reporting system, lack of awareness, and the perception that some events are not significant enough to report [25]. Additionally, inconsistent reporting standards and differences in reporting systems can lead to unreliable data collection, making it challenging to identify trends. Reports may also lack essential details, be inaccurate, or be incomplete, which hampers thorough analysis and effective interventions. Cumbersome reporting processes and a lack of feedback can discourage healthcare providers from reporting adverse events promptly. Cultural barriers that stigmatize errors and insufficient training on recognizing and reporting adverse events can also contribute to underreporting or misreporting. Furthermore, the lack of standardized definitions and protocols for reporting can lead to inconsistent data, hindering comparative analysis. Limited resources or expertise for a thorough analysis of reported events may result in missed opportunities to identify root causes and implement effective preventive measures. Inadequate feedback loops, causing failure to provide timely feedback to reporters and stakeholders about the outcomes of reported incidents, can diminish trust in the reporting system and discourage future reporting. Lastly, a culture of silence that discourages open discussion about errors and adverse events can impede transparency, hinder efforts to learn from mistakes, and prevent their recurrence.

Addressing these flaws requires a multifaceted approach, including promoting a culture of safety and transparency, providing education and support for reporting, standardizing reporting processes, ensuring robust analysis of reported incidents, and fostering a continuous feedback loop for improvement.

Key recommendations

Streamlining the adverse event reporting process in the emergency department will prompt more healthcare professionals to report incidents promptly. Offering comprehensive education and training programs for emergency department staff to heighten their awareness of adverse events and enhance their reporting capabilities. Introducing anonymous reporting options to address concerns about potential repercussions and nurturing a culture of openness and transparency in reporting adverse events. Establishing clear and standardized definitions of adverse events will ensure consistent understanding and reporting among all emergency department healthcare professionals. Utilizing technology to create a user-friendly electronic reporting system that facilitates quick and efficient submission of adverse event reports reduces paperwork and administrative burden. Establishing a feedback mechanism to communicate the outcomes of reported adverse events to healthcare professionals fosters a sense of accountability and learning from incidents. Convening multidisciplinary review panels to conduct thorough investigations of adverse events, analyze root causes, and develop targeted interventions to prevent future occurrences. Cultivating collaboration with regulatory bodies to align reporting frameworks with national or regional standards, ensuring consistency and compliance. Implementing recognition programs or incentives to motivate healthcare professionals to actively participate in reporting adverse events and acknowledge their valuable contributions to patient safety. Promoting a culture of continuous improvement by regularly reviewing and updating the adverse event reporting framework based on feedback, emerging trends, and advancements in healthcare practices.

Proactively managing adverse event reporting is essential to healthcare, particularly in the Emergency Department, so that healthcare providers can ensure that patients receive the best care, even in the most challenging circumstances. To achieve this, fostering a culture of open communication that emphasizes learning from incidents rather than blaming individuals is necessary. This can be achieved by implementing standardized reporting systems and conducting regular reviews to identify systemic issues. In addition, it is essential to perform root-cause analyses to understand the underlying causes of adverse events and identify areas for improvement. This might involve reviewing policies and procedures, implementing new training programs, or making changes to the physical environment of the Emergency Department.

Limitations

While this study has provided valuable insights into adverse event reporting frameworks and management approaches in healthcare, it is essential to acknowledge its limitations. The research conducted may have been limited due to several factors. The availability of data and access to relevant stakeholders may have been restricted, which could have resulted in specific healthcare settings, regions, or perspectives not being fully represented. This could potentially limit the generalizability of the findings. The study's reliance on existing literature and case studies may introduce biases or gaps in understanding, subject to the quality and breadth of the literature reviewed. The study's focus on specific healthcare settings may have overlooked unique challenges and opportunities in other healthcare contexts. The study's timeframe and resource constraints may have limited the depth of analysis or precluded longitudinal assessments of the effectiveness of implemented interventions, and the dynamic nature of healthcare systems, including healthcare workers' education, training, and skills, may have affected the relevance and applicability of the findings.

In summary, while this study contributes valuable insights into adverse event reporting frameworks and management approaches in healthcare, its limitations underscore the need for future research to build upon and address these constraints, ensuring a more comprehensive and nuanced understanding of this critical aspect of patient safety and quality improvement.

## Conclusions

The research states that satisfaction and dissatisfaction scores were nearly equal, suggesting underlying issues in healthcare services and the need for a more targeted approach to address specific concerns and enhance overall satisfaction. The research emphasizes the importance of developing a robust adverse event reporting framework and an effective management approach in healthcare systems to ensure patient safety and improve the quality of care. It highlights the need for standardized, transparent, and easily accessible reporting mechanisms and the significance of fostering a culture of open communication and accountability within healthcare organizations. The research also underscores the systematic approach to managing reported adverse events, including thorough investigation, root cause analysis, and implementing corrective actions. Leveraging technology and the role of regulatory bodies and accrediting agencies in promoting standardized adverse event reporting and management practices are also discussed.

The conclusion emphasizes the need for continued research, innovation, and collaboration to address emerging challenges and enhance patient safety and quality of care across healthcare systems. Additionally, it suggests the need for a large-scale survey to study the population's perceptions and expectations and the development of effective feedback mechanisms to provide insight into the prevailing safety culture and its improvement.

## Appendices

**Table 8:** Healthcare professional satisfaction survey questionnaire with information and consent

			SATISFACTION QUESTIONNAIRE																		
This questionnaire has been devised to give us your overall opinion of your care in the emergency department. It is not a test with no right or wrong answers. We are interested in your views and impressions, whether they are GOOD or BAD.																					
The participant can choose whether to participate or not.																					
The information provided by the respondent will be kept confidential and will be used only for research purposes.																					
The questionnaire consists of statements about care in the emergency department. Some statements may look the same, but they are different, so please read each one carefully before filling it. Please place a tick in the column that most closely resembles your opinion.																					
ONLY TICK ONE BOX FOR EACH STATEMENT																					
The example											No comment	Very poor	Poor		Good			Excellent			
Ambulance service and EMT skills before reaching the Emergency department.															√						
Please keep in mind that we are trying to find out your opinions, not those of your husband, wife, or neighbour, so please complete the questionnaire yourself.																					
Please try to think about the care you are receiving at the PRESENT TIME and give us your opinions.																					
The research will benefit the management by revealing various shortcomings in the policies, protocols, and procedures currently followed. There will be scope for improvement to increase the overall healthcare safety culture in the hospital.																					
		THANK YOU FOR YOUR HELP																			
													Signature of Surveyor								
						Survey / Questionnaire (Consent)															
I ............................................... (Participant name) understand that I am being asked to participate in a survey / Questionnaire. I have read the information above. I consent to participate in this survey/questionnaire project by signing the form below and returning it. Participant Signature																					
HEALTHCARE WORKER QUESTIONNAIRE Occupation: .........................................																					
											No comments	Very poor		Poor		Good			Excellent		
1	Healthcare adverse event (HAE) reporting framework for various errors																				
2	Management approach in handling healthcare adverse event reporting																				
