# Correction: Healthcare Worker’s Satisfaction Assessment for a Healthcare Adverse Event Reporting Framework and the Management Approach for Such Reporting in the Emergency Department of Rural Government Hospitals

**DOI:** 10.7759/cureus.c427

**Published:** 2026-04-30

**Authors:** Jyotsana Singh, Anita Choudhary, Somendra P Singh, Pankaj Singh

**Affiliations:** 1 Department of Management, NIMS University Rajasthan, Jaipur, IND; 2 Department of Management and Commerce, NIMS University Rajasthan, Jaipur, IND; 3 Department of General Surgery, Uttar Pradesh University of Medical Sciences, Etawah, IND; 4 Department of Anaesthesia, Uttar Pradesh University of Medical Sciences, Etawah, IND

This article has been corrected to change Dr. Jyotsana Singh’s affiliation to Department of Management, NIMS University Rajasthan, Jaipur, IND.

